# Impact of κ-Carrageenan on the Freshwater Mussel (*Solenaia oleivora*) Protein Emulsion Gels: Gel Formation, Stability, and Curcumin Delivery

**DOI:** 10.3390/gels10100659

**Published:** 2024-10-14

**Authors:** Wanwen Chen, Wu Jin, Xueyan Ma, Haibo Wen, Gangchun Xu, Pao Xu, Hao Cheng

**Affiliations:** 1 Key Laboratory of Freshwater Fisheries and Germplasm Resources Utilization, Ministry of Agriculture and Rural Affairs, Freshwater Fisheries Research Center, Chinese Academy of Fishery Sciences, Wuxi 214081, China; chenwanwen@ffrc.cn (W.C.);; 2 Wuxi Fisheries College, Nanjing Agricultural University, Wuxi 214081, China; 3 Sino-US Cooperative International Laboratory for Germplasm Conservation and Utilization of Freshwater Mollusks, Freshwater Fisheries Research Center, Chinese Academy of Fishery Sciences, Wuxi 214081, China; 4 State Key Laboratory of Food Science and Resources, School of Food Science and Technology, Jiangnan University, Wuxi 214122, China

**Keywords:** aquatic protein, κ-carrageenan, cold-set emulsion gel, encapsulation, in vitro digestion, bioaccessibility

## Abstract

Protein-based emulsion gels are an ideal delivery system due to their unique structure, remarkable encapsulation efficiency, and tunable digestive behavior. Freshwater mussel (*Solenaia oleivora*) protein isolate (SoPI), an emerging sustainable protein with high nutritional value, possesses unique value in the development of functional foods. Herein, composite emulsion gels were fabricated with SoPI and κ-carrageenan (κ-CG) for the delivery of curcumin. SoPI/κ-CG stabilized emulsions possessed a high encapsulation efficiency of curcumin with a value of around 95%. The addition of κ-CG above 0.50% facilitated the emulsion gel formation and significantly improved the gel strength with 1326 g. Furthermore, the storage and digestive stability of curcumin were significantly improved as the κ-CG concentration increased. At 1.50% κ-CG, around 80% and 90% curcumin remained after 21-day storage at 45 °C and the 6 h in vitro gastrointestinal digestion, respectively. The addition of 0.50% κ-CG obtained the highest bioaccessibility of curcumin (~60%). This study illustrated the potential of SoPI emulsion gels as a carrier for stabilizing and delivering hydrophobic polyphenols.

## 1. Introduction

Emulsion gels are a kind of soft, solid-like food system possessing both emulsion and gel characteristics that have been used for encapsulation, protection, and delivery of both hydrophilic and lipophilic bioactives [1,2]. Commonly used proteins for the fabrication of emulsion gels are milk proteins, plant proteins, and myofibrillar proteins [3,4,5]. The appearance, physicochemical properties, digestive behavior, and controlled release properties of emulsion gels are primarily determined by their structural type, material category and concentration, and intermolecular interplays [6,7,8,9]. Recently, the utilization of protein-polysaccharide interplays has become a more effective strategy for the fabrication and regulation of emulsion gels. Compared with emulsion gels formulated with a single protein, the existence of polysaccharides influenced not solely the gelation process and the architecture of the aqueous phase but also had an impact on the distribution of oil droplets, as well as the interfacial composition and structures [10,11,12]. Leveraging the combined benefits of polysaccharides and proteins, it becomes feasible to create emulsion gels with multi-level or hierarchical microstructures that exhibit improved stability and tunable digestive characteristics [8,13,14]. 

Recently, there has been a heightened interest in exploring novel edible and sustainable protein sources derived from aquatic animals and their by-products. This is attributed to their lower greenhouse gas emissions, fewer religious dietary restrictions, and reduced risks of immunogenic diseases compared to proteins obtained from terrestrial sources [15,16]. *Solenaia oleivora*, a significant freshwater economic shellfish found exclusively in China, boasts a low-fat content and is particularly rich in protein, accounting for 73% of its dry weight in the edible parts [17]. Freshwater mussel (*Solenaia oleivora*) protein isolate (SoPI) possesses a high content of leucine, lysine, valine, and isoleucine lysine and a high essential amino acid index of 49.2, making it suitable for the development of functional food products [18]. Despite the poor hydrosolubility and functionality of SoPI, our previous studies have demonstrated processing treatment (e.g., ultrasound and high-pressure homogenization) can significantly reduce particle size and enhance hydrophobicity and surface charges, resulting in the improvement of hydrosolubility and emulsifying activities of SoPI [18,19]. However, SoPI is currently underutilized in the fabrication of emulsions and gels. Hence, it is imperative to explore viable processing techniques aimed at enhancing the properties of SoPI and broadening its application spectrum within food products.

Curcumin, a natural polyphenolic compound, possesses a bis-feruloylmethane structure with a range of beneficial effects, including antioxidant, anti-inflammatory, antibacterial, anticancer, liver-protective, and anti-atherosclerosis effects [20]. However, natural curcumin suffers from poor hydrosolubility, sensitivity to environmental factors including oxygen, light, and heat, and diminished bioavailability after oral administration, thereby restricting its application in functional foods [21,22]. Consequently, the development of edible delivery systems is essential to improve their physicochemical stability and bioaccessibility. To date, emulsion gels fabricated with numerous proteins (e.g., whey proteins, pork myofibrillar protein, pea proteins, and rice proteins) have been reported for the encapsulation and protection of curcumin and regulation of gastrointestinal digestion behavior [23,24,25,26]. However, there are no reported studies focused on the fabrication of emulsion gels formulated with SoPI for the delivery of curcumin. 

κ-Carrageenan (κ-CG), a linear polysaccharide, consists of α-(1-3)-D-galactopyranose and β-(1-4)-3,6-anhydro-D-galactopyranose with 15–40% ester sulfate content [27]. κ-CG/protein composite gel networks can be induced by salt ions, acids, or spontaneously formed at room temperature after heating. The presence of κ-CG in protein-based emulsion gels can improve protective effects and controlled release of bioactives, which is dependent on the protein/polysaccharide interplays, polysaccharide concentration, and gelation methods. [28,29,30,31]. Increasing κ-CG concentration is an effective strategy to induce protein micro-phase separation or protein/κ-CG segregative phase separation, resulting in the formation of protein/κ-CG coupled gel networks or κ-CG dominated gel networks entrapped protein phase or emulsified oil droplets [32,33]. The diverse gel structures facilitate the modification of gel texture and controlled hydrolysis of protein and oil phase during the digestion process [1]. For example, a low κ-CG concentration at 0.25% could enhance the lipid hydrolysis of pea protein emulsion gels during the digestion due to decreased oil droplet aggregation and coalescence, while a high κ-CG concentration above 1.0% could significantly delay the digestion of pea protein and oil phase because of stronger gel networks, higher viscosity, and more extensive flocculation [10]. Su and co-workers reported that the incorporation of κ-CG in curcumin-loaded whey protein emulsion gels could decrease the release of curcumin during the gastric phase, leading to more curcumin released in the intestinal phase [23]. 

This study aims to fabricate the medium-chain triglyceride (MCT) oil emulsion gel formulated with SoPI and κ-CG for the delivery of curcumin. Impacts of the κ-CG on texture properties of SoPI emulsion gel and chemical stability, release profile, and bioaccessibility of curcumin were explored. Our research aims to expand the utilization of SoPI as a novel protein source, paving the way for the development of edible delivery systems within the food industry. 

## 2. Results and Discussion

### 2.1. Emulsion Characterization

#### 2.1.1. Particle Size Distribution and ζ-Potential

**Figure 1:**
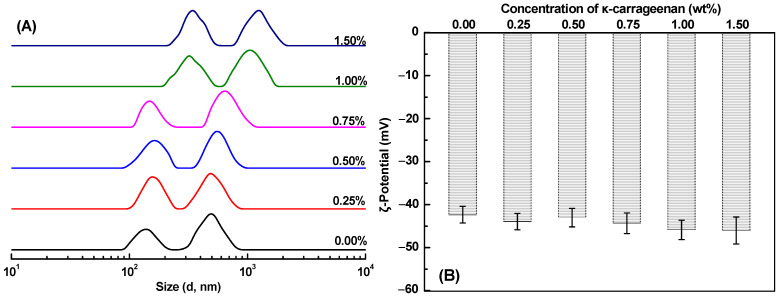
Effect of κ-carrageenan concentration on the size distribution (**A**) and ζ-potential (**B**) of SoPI-stabilized emulsions.

As shown in Figure 1A, all SoPI-stabilized emulsions with or without κ-CG showed a bimodal size distribution. For SoPI-stabilized emulsions, the smaller peak ranging from 100 to 200 nm was attributed to the SoPI aggregates [18], while the peak above 500 nm was ascribed to the oil droplets [34]. The particle sizes of both SoPI aggregates and emulsified oil droplets increased slightly with increasing κ-CG concentration. At low polysaccharide concentrations, both negatively charged κ-CG and SoPI molecules may co-solubilize in the natural environment, thereby exhibiting no significant impact on the emulsified oil droplets [35]. The particle size of the emulsified oil droplets varied from 1 μm to 2 μm as the κ-CG concentration was above 1.0%, possibly resulting from the depletion flocculation between emulsified oil droplets induced by the presence of much unabsorbed κ-CG molecules in the continuous phase [36,37]. In this situation, the presence of unadsorbed κ-CG molecules within the continuous phase induces an osmotic gradient, which results in the extraction of water from the droplet’s depletion region, ultimately causing aggregation of the oil droplets [38].

The ζ-potential of SoPI emulsions was about −42 mV (Figure 1B). The pH of the emulsions at around 7.0 was higher than the isoelectric point of SoPI (about 4.5~5.5) [39], and thus emulsified oil droplets exhibited negatively charged surfaces. The addition of κ-CG exhibited no significant impact (*p* < 0.05) on the ζ-potential of emulsified oil droplets, with values ranging from −42 to −46 mV. It can be inferred that κ-CG was not adsorbed onto the surface of the emulsified oil droplets at pH 7.0 due to the relatively large electrostatic repulsion between negatively charged κ-CG containing sulfate groups and emulsified oil droplets [37]. The above results indicated that the negatively charged κ-CG molecules were mainly presented in the aqueous phase of emulsions [36,40].

#### 2.1.2. Encapsulation Efficiency of Curcumin in Emulsions

**Figure 2:**
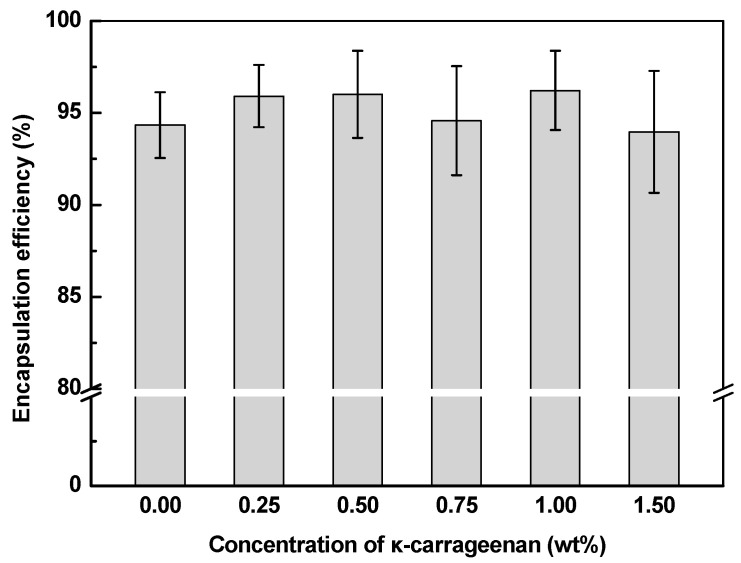
Encapsulation efficiency of curcumin in SoPI-stabilized emulsions at various κ-carrageenan concentrations.

Curcumin possesses a high log *p* value of 3.29 and limited solubility in aqueous solution with a value of 11 ng/mL at ambient temperature [21]. Encapsulation within protein particles and emulsions is an effective strategy for improving the hydrosolubility and stability of curcumin [41,42]. In Figure 2, the encapsulation efficiency of curcumin in SoPI emulsions was around 94%, which is similar to the Tween 80/Span 80 stabilized MCT emulsions (87–98%) [41] and glutelin fibrils stabilized MTC emulsions (~94%) [43]. The relatively high encapsulation efficiency may result from the good emulsifying properties of ultrasound treatment SoPI and strong interfacial protein membrane formation, preventing the release of curcumin from the oil phase to the aqueous phase [18,44]. Furthermore, the lipophilic nature of curcumin made it more likely to be located in the inner oil phase of emulsions [45]. The addition of κ-CG had no significant impact on the curcumin encapsulation. These results suggest that SoPI emulsions could be as good curcumin-loaded carriers.

### 2.2. Emulsion Gel Formation and Characterization

#### 2.2.1. Visual Appearance

**Figure 3:**
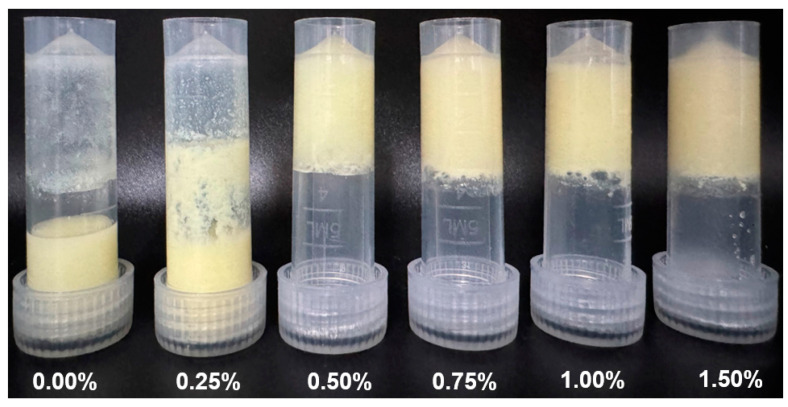
Appearance image of curcumin-loaded SoPI emulsion gels with different κ-carrageenan concentrations.

Figure 3 shows the visual appearance of the inverted curcumin-loaded SoPI emulsion gels with κ-CG. SoPI emulsions with or without 0.25% κ-CG flowed to the tube bottom, suggesting emulsions at low protein concentrations (1% *w*/*w*) could not form the self-support gel structure. Conversely, the self-support emulsion gel formation could be observed as the κ-CG concentration was above 0.50%. Therefore, SoPI emulsion gels with κ-CG varied from 0.50% to 1.50% and were chosen for subsequent characterization.

#### 2.2.2. Water Holding Capacity

**Figure 4:**
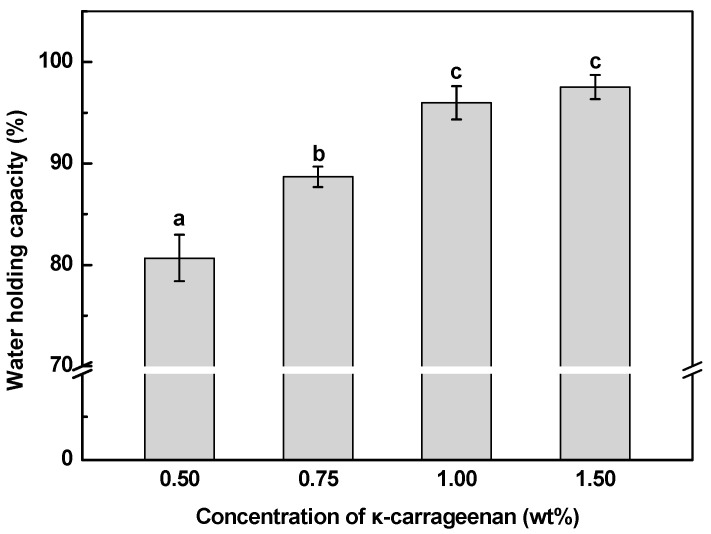
Effects of κ-carrageenan concentration on the water-holding capacity of SoPI emulsion gels. Different letters indicate the statistically significant difference (*p* < 0.05).

Generally, the water-holding capacity of SoPI emulsion gels is expected to increase with increasing κ-CG concentrations (Figure 4). As the κ-CG concentration was above 1.00%, the water-holding capacity of SoPI emulsion gels reached 96%. The elevated concentration of κ-CG promoted the formation of SoPI/κ-CG composite or continuous κ-CG dense gel networks with diminished pore sizes, which in turn enhanced stronger capillary forces, facilitating the retention of a greater number of water molecules [10]. Moreover, the substantial number of hydroxyl groups on the κ-CG can engage in hydrogen bonding with water molecules, thereby contributing to a reduction in water loss [46,47].

#### 2.2.3. Textural Properties

Table 1 shows the texture characteristics of SoPI emulsion gels with various concentrations of κ-CG. Overall, the textural profile of emulsion gels was mainly determined by the structure of the gel matrix and emulsified oil droplets and their interactions [6]. The gel hardness and chewiness increased with increasing κ-CG concentration, with values of 1326 g and 169 g at 1.50% κ-CG, respectively. This indicates that the incorporation of κ-CG could improve the mechanical properties of SoPI emulsion gels. A similar phenomenon was also observed in the pea protein/κ-CG composite emulsion gels [31]. Polysaccharides exhibit thickening and space-occupying effects on the protein gel formation process [48]. The increased κ-CG composite would induce the formation of the carrageenan continuous gel matrix with high water content (Figure 4) and decreased void spaces, thereby increasing gel strength [32,49]. Furthermore, increasing polysaccharide content may facilitate interplays between SoPI-coated oil droplets and κ-CG in an acidic environment, contributing to the further increase in gel strength. The addition of κ-CG slightly increased the gel springiness but did not significantly affect the cohesiveness of SoPI emulsion gels (Table 1). This observation is consistent with findings in emulsion gels formulated with egg yolk and sodium alginate [50] and curcumin-loaded whey protein/sugar beet pectin composite emulsion gels [49]. 

### 2.3. Chemical Stability of Curcumin in the Emulsion and Emulsion Gel

**Figure 5:**
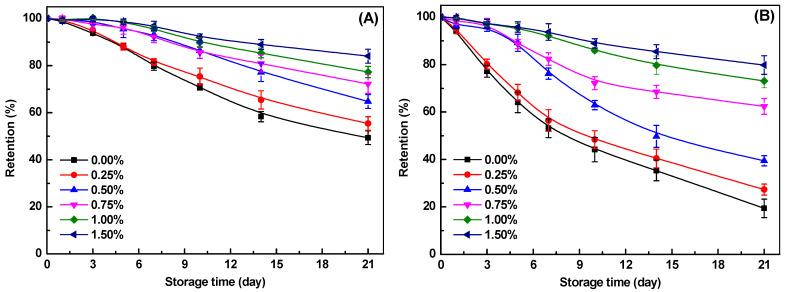
Retention of curcumin encapsulated within SoPI emulsion gels at various κ-carrageenan concentrations during storage for 21 days at 20 °C (**A**) and 45 °C (**B**).

Curcumin is highly unstable in neutral and alkaline conditions and labile to degradation under light and heat [51,52]. It has been reported that curcumin dispersed in the aqueous phase experienced a degradation of over 80% during 30 min at pH 5.0, due to the autoxidative process driven by free radicals [22,52]. In Figure 5A,B, the retention of curcumin in the SoPI emulsions decreased slowly during the storage both at 20 °C and 45 °C, with 49% and 19% remaining after 21 days, respectively. The high retention of curcumin in SoPI emulsions is because curcumin was encapsulated within the inner oil phase, which afforded it substantial protection from the sensitive external environment [53]. Furthermore, the decreased pH during the emulsion gel formation induced by glucono-δ-lactone (GDL) could further improve the physicochemical stability of curcumin molecules [52]. 

Following the addition of κ-CG, the degradation of curcumin was further diminished (Figure 5), indicating that the emulsion gel structure offered robust protection for curcumin. This protective effect becomes more pronounced at high κ-CG concentrations, both at 20 °C and 45 °C. As the κ-CG concentration was 1.50%, the retention of curcumin was around 84% and 80% after storage at 20 °C and 45 °C for 21 days, respectively (Figure 5). Previous studies demonstrated that bioactives entrapped within a gel network could improve their physicochemical stability [2,54]. The dense emulsion gel networks, characterized by high mechanical properties, were expected to be more effective in retarding the degradation of encapsulated bioactives [51]. This may be attributed to the effective physical barrier that prevents the diffusion of pro-oxidants or free radicals [55]. Furthermore, the hard gel networks could restrict the mobility of bioactive molecules and contact with environmentally sensitive agents during storage [23]. Therefore, the encapsulation of curcumin in SoPI/κ-CG composite emulsion gels with high κ-CG concentration and strong mechanical properties (Table 1) could effectively improve its chemical stability during storage.

### 2.4. In Vitro Digestion

#### 2.4.1. Free Fatty Acids Release

**Figure 6:**
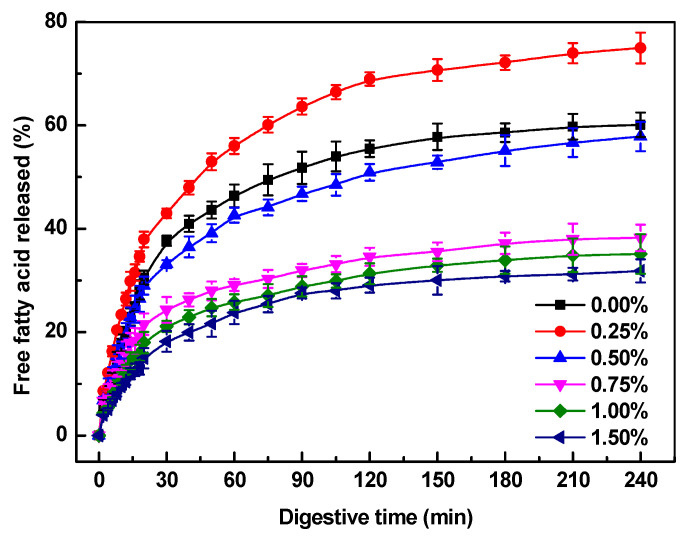
The release of free fatty acid from SoPI emulsion gels at various concentrations of κ-carrageenan during in vitro intestinal digestion.

Figure 6 illustrates the release of free fatty acids from SoPI emulsion gels at different concentrations of κ-CG throughout the intestinal digestion process. Generally, there was a swift surge in free fatty acids at the onset, which then tapered off to a more moderate increase over time. Without the presence of κ-CG, approximately 38% of free fatty acids were liberated within the first 30 min. Subsequently, the free fatty acid release decelerated, reaching a total of 60% by the conclusion of the intestinal digestion phase.

The inclusion of 0.25% κ-CG enhanced both the rate and extent of free fatty acid release throughout the digestion process, with values of 43% and 75% after 30 min and 240 min, respectively (Figure 6). This accelerated effect may be attributed to anionic κ-CG coated on the surface of SoPI emulsified oil droplets preventing the massive aggregation of oil droplets in the gastric phase, thereby facilitating the adsorption of lipases onto the oil droplets and lipid hydrolysis [56]. As the κ-CG concentration was above 0.50%, the production of free fatty acids from emulsion gels began to slow down. Notably, at a 1.5% κ-CG concentration, there was a significant suppression of lipid digestion, with only 18% and 32% of lipids being digested after 30 min and 240 min, respectively. The decrease in lipid hydrolysis at high κ-CG concentration was mainly attributed to the hard and dense gel networks that prevent the disintegration of the gel matrix and digestive enzymes from accessing the gel interior during intestinal digestion [57]. Moreover, the elevated viscosity and extensive flocculation and coalescence induced by cations present in the gastrointestinal fluids may result in diminished accessibility for lipase to the oil droplets [58]. 

#### 2.4.2. In Vitro Digestive Stability

**Figure 7:**
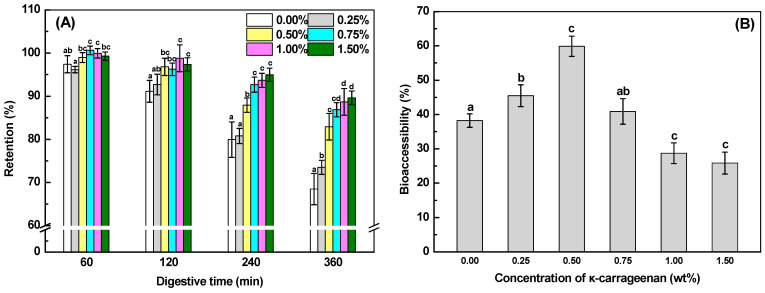
Retention (**A**) and bioaccessibility (**B**) of curcumin in SoPI emulsion gels at various concentrations of κ-carrageenan during in vitro digestion. Different letters indicate the statistically significant difference (*p* < 0.05).

As shown in Figure 7A, all samples have a curcumin retention of above 90% after the gastric digestion stage. It is to be expected that the significantly reduced contents of curcumin could be observed during the small intestine digestion since curcumin was rapidly degraded to bicyclopentadione, ferulic acid, and vanillin at neutral and alkaline conditions [59]. The degradation of curcumin was reduced in the small intestine stage as the κ-CG concentration increased. The retention of curcumin was above 83% after the overall digestion, as the κ-CG concentration was higher than 0.50%. These results demonstrated that the incorporation of κ-CG into SoPI emulsion gels could enhance the digestive stability of curcumin. This result was in line with curcumin-loaded composite emulsion gel with myofibrillar protein and carboxymethyl cellulose [24]. The enhanced protective effects may be due to the stronger gel structure (Table 1) and the less release of curcumin-loaded oils to the digestive fluids (Figure 6) [60].

#### 2.4.3. Bioaccessibility of Curcumin

Water-dispersed curcumin possesses a low bioaccessibility varied from 2% to 17% due to its relatively high Log *p* value of 3.29 and chemical instability [41,61]. The hydrolysis of lipids during digestion promotes the formation of mixed micelles stabilized by bile salts, leading to the solubilization of curcumin and improving its bioaccessibility [62]. Therefore, the bioaccessibility of curcumin loaded within the oil phase of emulsion gels was largely influenced by lipid digestion. The bioaccessibility of curcumin in SoPI emulsions was 38% (Figure 7B). The addition of κ-CG at 0.25% could further increase the bioaccessibility of curcumin to 45%. With the further increase in κ-CG concentration to 0.50%, the bioaccessibility of curcumin reached its highest level with a value of around 60%. It is important to mention that the bioaccessibility of curcumin was not in proportion to the amount of free fatty acids, as the κ-CG concentration varied from 0% to 0.50%. The reason for the lower bioaccessibility of curcumin at 0% and 0.25% κ-CG may be due to the relatively faster degradation (Figure 7A) and insufficient micelle production for the solubilization of initially and rapidly released curcumin molecules. However, the high κ-CG concentration varied from 1.00% to 1.50%, resulting in a significant decrease in the bioaccessibility of curcumin with values of 29% and 26%, respectively (Figure 7B). The reduced bioaccessibility could be largely attributed to the substantial amount of curcumin that persisted within the undigested oil droplets of the emulsion gel system following exposure to simulated intestinal digestion (Figure 6). 

## 3. Conclusions

In this study, emulsion gels formulated with SoPI and κ-CG have been successfully fabricated, exhibiting good encapsulation, protection, and delivery performance for curcumin. The addition of κ-CG could facilitate the SoPI emulsion gel formation and improve the gel strength and protective effect of encapsulated curcumin against degradation during the storage and in vitro digestion processes. Furthermore, κ-CG concentration had a significant impact on the in vitro digestion behavior of emulsions and the bioaccessibility of curcumin. A middle-level κ-CG concentration at 0.50% facilitates achieving the highest bioaccessibility of curcumin in the SoPI emulsion gels. These findings obtained here should provide an understanding of SoPI for the development of emulsion gels as effective carriers for hydrophobic polyphenols.

## 4. Materials and Methods

### 4.1. Materials

Fresh freshwater mussel (*Solenaia oleivora*) was obtained by Jinghuai Special Aquatic Products Co., Ltd. (Funan, China). Medium-chain triglyceride (MCT) oil (C8:C10 = 60:40) was obtained from Yong Sheng Industry and Trade Co., Ltd. (Guangzhou, China). Porcine pepsin (≥500 U/mg), bile salts, porcine pancreatin (4 × USP specifications), κ-carrageenan, and curcumin were obtained from Sigma-Aldrich Co., Ltd. (St. Louis, MO, USA). Other analytical grade agents were provided by SinoPharm CNCM Ltd. (Shanghai, China).

### 4.2. Freshwater Mussel (Solenaia oleivora) Protein Isolation and Ultrasound Treatment

Freshwater mussel (*Solenaia oleivora*) protein was isolated using an alkaline extraction–isoelectric precipitation method and then treated by ultrasonication according to our previously reported study [18]. Briefly, shelled and mashed mussel flesh was mixed with water at a weight ratio of 1:6 at pH 12.0 for 1 h using 4 M NaOH. This mixture was then centrifuged at 10,000× *g* for 20 min at 20 °C. The pH of the resulting supernatant was adjusted to 5.0 using 2 M HCl and left for 30 min before being centrifuged at 10,000× *g* for 10 min. The precipitate was redispersed and neutralized to a pH of 7.0 using 2 M NaOH. The protein dispersion was frozen at −20 °C overnight and then lyophilized at −80 °C/0.014 mBar for 72 h by a benchtop freeze dryer (Freezone, 2.5, Labconco, Kansas City, MO, USA). The obtained protein powder was stored at −20 °C. The purity of the final SoPI powder was assessed to be 95.2% using the Kjeldahl method.

Pre-cooled SoPI dispersions at 2.0 wt% were processed using ultrasonication at an output power of 600 W (131–138 W/cm^2^) for 20 min with a pulse cycle consisting of 5 s on-time and 1 s off-time using an ultrasonicator (Jingping Instrument Co. Ltd., Wuxi, China). The SoPI samples that underwent ultrasonic treatment were stored at 4 °C for further use. 

### 4.3. Emulsion Preparation

κ-CG (3%, *w*/*w*) stock solution was prepared by dispersing the powder into deionized water while stirring continuously at a temperature of 55°C. Curcumin was dispersed in MCT at 1 mg/mL. To prepare the MCT emulsions, the ultrasound-treated SoPI solution was combined with MCT at a 5% (*w*/*w*) concentration, using a high-speed blender set at 12,000 rpm for 2 min. This mixture then underwent high-pressure homogenization at 45 MPa for 4 cycles. Subsequently, varying amounts of the κ-CG solution were introduced to the SoPI-stabilized emulsions to produce SoPI/κ-CG emulsions. The resulting emulsions comprised 1% (*w*/*w*) ultrasound-treated SoPI and an κ-CG concentration ranging from 0 to 1.5% (*w*/*w*) in the aqueous phase. Sodium azide (0.02%, *w*/*v*) was added to the final emulsions for the inhibition of microorganism growth.

### 4.4. Particle Size Distribution and ζ-Potential Measurement

Emulsions were diluted with ultrapure water. Their particle sizes were then assessed three times at 25 °C with a scattering angle of 90° using a NanoBrook Omni particle size analyzer (NanoBrooker Omni, Brookhaven Instruments Ltd., New York, NY, USA). The ζ-potential values were derived from calculations based on the Smoluchowski theory.

### 4.5. Encapsulation Efficiency of Curcumin in the Emulsion

The content of curcumin distributed in emulsified oil droplets of emulsions was detected by previously reported protocols [25]. Emulsions were subjected to centrifugation with a 5804 R centrifuge (Eppendorf Co., Ltd., Hamburg, Germany) at 13,000× *g* for 30 min at 4 °C. The whole emulsion and the obtained aqueous phase were mixed with a 9-fold volume of ethanol, followed by a centrifugation at 10,000× *g* for 10 min at 4 °C. The content of curcumin in the ethanol extracts was determined by a UV–visible spectrophotometer (Shimadzu, Tokyo, Japan) at 425 nm according to a curcumin standard curve at concentrations ranging from 0.25 μg/mL to 10 μg/mL. The background of the blank sample was subtracted from raw experimental data. The encapsulation efficiency was calculated utilizing Equation (1):

(1) $$\mathrm{Encapsulation}\;\mathrm{efficiency}\;(\%)=\left(1-\frac{C_{a}}{C_{t}}\right)\;\times \;100\%$$

where *C*_t_ and *C*_a_ were the content of curcumin in the whole emulsion and the aqueous phase, respectively. 

### 4.6. Emulsion Gel Fabrication

Emulsion gels were fabricated with glucono-δ-lactone (GDL) following previously reported protocols [10]. The emulsions were rapidly mixed with 25% GDL and stored overnight at 4 °C for gel formation. The final concentration of GDL was 1% in the emulsion gels. The final pH of emulsion gels was around 4.85.

### 4.7. Water Holding Capacity Analysis

The water-holding capacity of emulsion gels was carried out following a previously established protocol [48]. Approximately 3 g of the gel sample was transferred into a 50 mL tube, which was then subjected to centrifugation at a speed of 8000× *g* for 20 min. Following this process, any surplus water was carefully blotted away using filter paper. The water holding capacity was subsequently calculated utilizing Equation (2):

(2) $$\mathrm{Water}\;\mathrm{holding}\;\mathrm{capacity}\;(\%)=\left(1-\frac{W_{t}-W_{e}}{W_{t}}\right)\;\times \;100\%$$

where *W*_t_ symbolizes the mass of the emulsion gel before centrifugation (expressed in grams), while *W*_e_ represents the mass of the emulsion gel after it has been centrifuged to remove excess water (also in grams).

### 4.8. Texture Profile Analysis

Texture properties were characterized by a TA. XTPlus texture analyzer (Stable Micro Systems, Surrey, UK) equipped with a P-36 R cylindrical test probe (diameter = 36 mm) [10]. Cylindrical samples of the emulsion gel, measuring 2 cm in diameter and 1.5 cm in height, were positioned on the carrier table, which was conducted using a 3 g trigger force, 2-cycle sequence, a 50% strain, and a test speed of 1.0 mm/s. The texture analysis software supplied with the instrument generated the textural parameters.

### 4.9. Stability of Curcumin in Emulsion Gels

All samples were stored in an incubator at 20 °C and 45 °C for up to 21 days. The content of curcumin in emulsion gels was determined by previously reported protocols [25,63]. Briefly, 0.5 g of emulsion gel samples were mixed with 9.5 mL of ethanol for the curcumin recovery, and then the mixture was centrifuged at 10,000× *g* for 10 min. The resulting supernatant was determined by a UV spectrophotometer at 425 nm. The stability of curcumin within the whole emulsion gels was assessed by its retention throughout storage, which was quantified as a percentage relative to the initial curcumin content in freshly prepared emulsion gels.

### 4.10. In Vitro Digestion Analysis

#### 4.10.1. Simulated Gastrointestinal Digestion

The digestive behavior of emulsion gels was evaluated through an INFOGEST model with slight modifications [10,64]. Briefly, the emulsion gel sample (10 g) was shredded and then mixed with 10 mL simulated gastric fluid (pH 3.0, 2000 U/mL pepsin) for 2 h. The gastric digesta was further incubated with 20 mL of simulated intestinal fluid consisting of 100 U/mL pancreatin and 10 mM bile at pH 7.0 for 4 h. NaOH (0.1 M) was used to consistently maintain the pH of the digestion system at 7.0 throughout the entire simulated intestinal digestion process.

#### 4.10.2. Free Fatty Acid Measurement

Free fatty acid release during intestinal digestion was determined using the pH-stat method [65]. The pH of the intestinal digestive fluid was examined for different digestion times, while the pH was maintained at 7.0 by titration using NaOH solution. It is assumed that two free fatty acids are produced for each triacylglycerol molecule by lipase action. The release of free fatty acids was calculated using Equation (3):

(3) $$\mathrm{Free}\;\mathrm{fatty}\;\mathrm{acid}\;\mathrm{release}\;(\%)=\frac{V_{\mathrm{NaOH}}\;\times {\;C}_{\mathrm{NaOH}}\;\times {\;M}_{\mathrm{Lipid}}}{{2\;\times \;W}_{\mathrm{Lipid}}}\;\times \;100\%$$

V_NaOH_ is the consumed volume of NaOH for the titration (mL), C_NaOH_ is the molar concentration of NaOH (0.1 M), M_Lipid_ is the average molecular weight of MCT (500 g/mol), and W_Lipid_ is the weight of the lipid initially present in the reaction vessel (g). 

#### 4.10.3. Stability and Bioaccessibility of Curcumin during the Digestion

To assess the stability of curcumin, digesta were collected at predetermined intervals during digestion and analyzed as outlined in Section 4.9. Regarding bioaccessibility, samples were gathered following the completion of simulated intestinal digestion. These samples were then centrifuged at 10,000× *g* for 30 min at 4 °C. The resultant clear middle layer, presumed to be the micellar fraction containing solubilized curcumin, was isolated for analysis. The bioaccessibility of curcumin was determined using Equation (4).

(4) $$\mathrm{Bioaccessibility}\;(\%)=\frac{C_{\mathrm{micelle}}}{C_{\mathrm{total}}}\;\times \;100\%$$

where C_micell_ represents the quantity of curcumin solubilized within the micellar fraction, and C_total_ denotes the initial content of curcumin present in the emulsion gels.

### 4.11. Statistical Analysis

All measurements were taken in triplicate and reported as the mean value ± standard deviation. For statistical analysis, one-way analysis of variance (ANOVA) with the Duncan post hoc test was selected using SPSS software (SPSS 20.0, IBM SPSS Institute, Inc., New York, NY, USA). A *p* < 0.05 level was considered as the significant difference.

## Figures and Tables

**Table 1:** Effects of κ-carrageenan concentration on the mechanical characteristics of SoPI emulsion gels.

κ-Carrageenan Concentration (%)	Hardness (g)	Springiness	Cohesiveness	Chewiness (g)
0.50	516.8 ± 93.4 ^a^	0.489 ± 0.021 ^a^	0.359 ± 0.026 ^a^	88.91 ± 8.36 ^a^
0.75	689.1 ± 53.1 ^b^	0.510 ± 0.024 ^ab^	0.361 ± 0.021 ^a^	139.38 ± 7.56 ^b^
1.00	910.7 ± 81.3 ^c^	0.545 ± 0.023 ^b^	0.386 ± 0.023 ^a^	155.18 ± 15.32 ^bc^
1.50	1326.3 ± 108.3 ^d^	0.563 ± 0.039 ^b^	0.373 ± 0.031 ^a^	169.32 ± 19.16 ^c^

Different letters in the same column indicate statistically significant differences (*p* < 0.05).

## Data Availability

The data presented in this study are available on request from the corresponding author.
